# Zero anticoagulation, zero thrombolysis: successful management of massive pulmonary embolism following hemorrhagic transformation of acute ischemic stroke: a case report

**DOI:** 10.3389/fcvm.2026.1747104

**Published:** 2026-02-02

**Authors:** Zengkai Xu, Bing Ding, Jiahuang Wu, Hongjin Wang, Zeping Chen, Zhisheng Wang

**Affiliations:** 1 Division of Cardio-Thoracic Surgery, Longyan First Affiliated Hospital of Fujian Medical University, Longyan, Fujian, China; 2 Division of Pulmonary Medicine, Longyan First Affiliated Hospital of Fujian Medical University, Longyan, Fujian, China

**Keywords:** acute ischemic stroke, case report, hemorrhagic transformation, pulmonary embolism, zero anticoagulation, zero thrombolysis

## Abstract

**Background:**

The treatment of patients with severe pulmonary embolism complicated by intracerebral hemorrhage is challenging: anticoagulation or thrombolysis, essential for treating pulmonary embolism, can substantially worsen intracranial hemorrhage and threaten life.

**Case presentation:**

We report a case of a 78-year-old female who developed hemorrhagic transformation following acute ischemic stroke, complicated by massive pulmonary embolism and rapidly progressing to shock. In this case, emergent percutaneous mechanical thrombectomy was successfully performed under a zero-anticoagulation and zero-thrombolysis strategy, resulting in hemodynamic stabilization.

**Conclusion:**

This case provides valuable evidence supporting the feasibility of purely mechanical percutaneous intervention in high-risk, complex clinical scenarios.

## Introduction

Ischemic stroke is usually caused by arterial occlusion in the central nervous system and remains one of the leading causes of death and disability worldwide. Hemorrhagic transformation (HT), defined as the extravasation of blood into ischemic tissue, is a serious complication that worsens outcomes and increases mortality (1). Notably, some studies suggest that hemorrhagic transformation within the infarcted area represents a natural pathophysiological process (2). For patients with HT, continuing anticoagulation therapy may be reasonable depending on the specific clinical context and indications for anticoagulation (3). However, when intracranial hemorrhage shows a clear “progressive”pattern, any form of anticoagulation or thrombolytic therapy is generally considered absolutely contraindicated, as it may precipitate catastrophic symptomatic bleeding. Currently, clinical guidelines lack explicit recommendations regarding whether and when to resume antithrombotic therapy in patients with hemorrhagic transformation, particularly when the bleeding is still evolving. This gap in guidance leaves clinical decision-making largely dependent on individualized risk assessment.

This report presents a highly challenging and critical case. A 78-year-old woman with a history of atrial fibrillation on long-term apixaban therapy developed acute middle cerebral artery occlusion. She underwent successful percutaneous mechanical thrombectomy, but subsequently experienced progressive hemorrhagic transformation within the infarcted region. In response, all anticoagulant medications were immediately discontinued. Paradoxically, during this period of strict antithrombotic avoidance mandated to prevent hemorrhage progression, the patient suffered a massive acute pulmonary embolism, plunging her into a profound therapeutic dilemma between “progressive intracranial hemorrhage” and “fatal systemic thromboembolism.”Conventionally, the cornerstone treatment for pulmonary embolism—systemic anticoagulation or thrombolysis—was absolutely contraindicated in this scenario due to the presence of active intracranial bleeding. Faced with this impasse, a multidisciplinary team formulated and successfully executed a fully percutaneous, purely mechanical, life-supporting strategy without the use of any anticoagulants or thrombolytics (“zero anticoagulation, zero thrombolysis”). The pulmonary thrombus was effectively removed through mechanical aspiration thrombectomy, followed by the placement of an inferior vena cava filter to prevent recurrent embolization. This case provides an innovative therapeutic paradigm and decision-making framework for managing such extreme situations in the absence of guideline-based recommendations, where two life-threatening risks coexist.

## Case presentation

A 78-year-old woman presented to our emergency department with acute speech disturbance and left-sided weakness. The onset was unwitnessed. Her last known well time was approximately 6 h prior to arrival. She was found by her family already collapsed and symptomatic at home about 10 min before presentation. Her past medical history included hypertension, atrial fibrillation, diabetes mellitus, and previous ischemic stroke. She had been on long-term oral anticoagulation therapy with apixaban. A non-contrast head CT scan at a local hospital revealed right frontotemporal infarction. Because of the contraindication to thrombolytic therapy due to chronic apixaban use, she was transferred to our hospital for further management.

On admission, her vital signs were: temperature 36.3℃, pulse 68 bpm, respiratory rate 20 breaths/min, and blood pressure 85/61 mmHg. It is important to note that this relative hypotension was iatrogenic, secondary to the administration of intravenous urapidil for blood pressure control prior to the definitive diagnosis of stroke. The patient had a known history of hypertension.She was alert but had slurred speech. Pupils were equal and reactive to light (2.5 mm in diameter). Both eyes deviated to the right. The left nasolabial fold was shallow, tongue deviated to the left, and gag reflex was present and symmetrical. Muscle strength was grade 0 in the left upper limb and grade 2 in the left lower limb, while grade 5 in the right limbs. Muscle tone was normal. Tendon reflexes were symmetrical, Babinski sign was positive on the left and negative on the right. Neck was supple, and Kernig's sign was negative. Emergency cerebral angiography confirmed right middle cerebral artery (M1 segment) occlusion with a D-dimer level of 0.20 mg/L. Post-procedural imaging assessment estimated the volume of the ischemic stroke to be approximately 194.715 cm^3^.The patient underwent percutaneous mechanical thrombectomy, achieving successful vascular recanalization. Postoperatively, she received anticoagulation (low-molecular-weight heparin/rivaroxaban) and supportive care.

**Figure 1:**
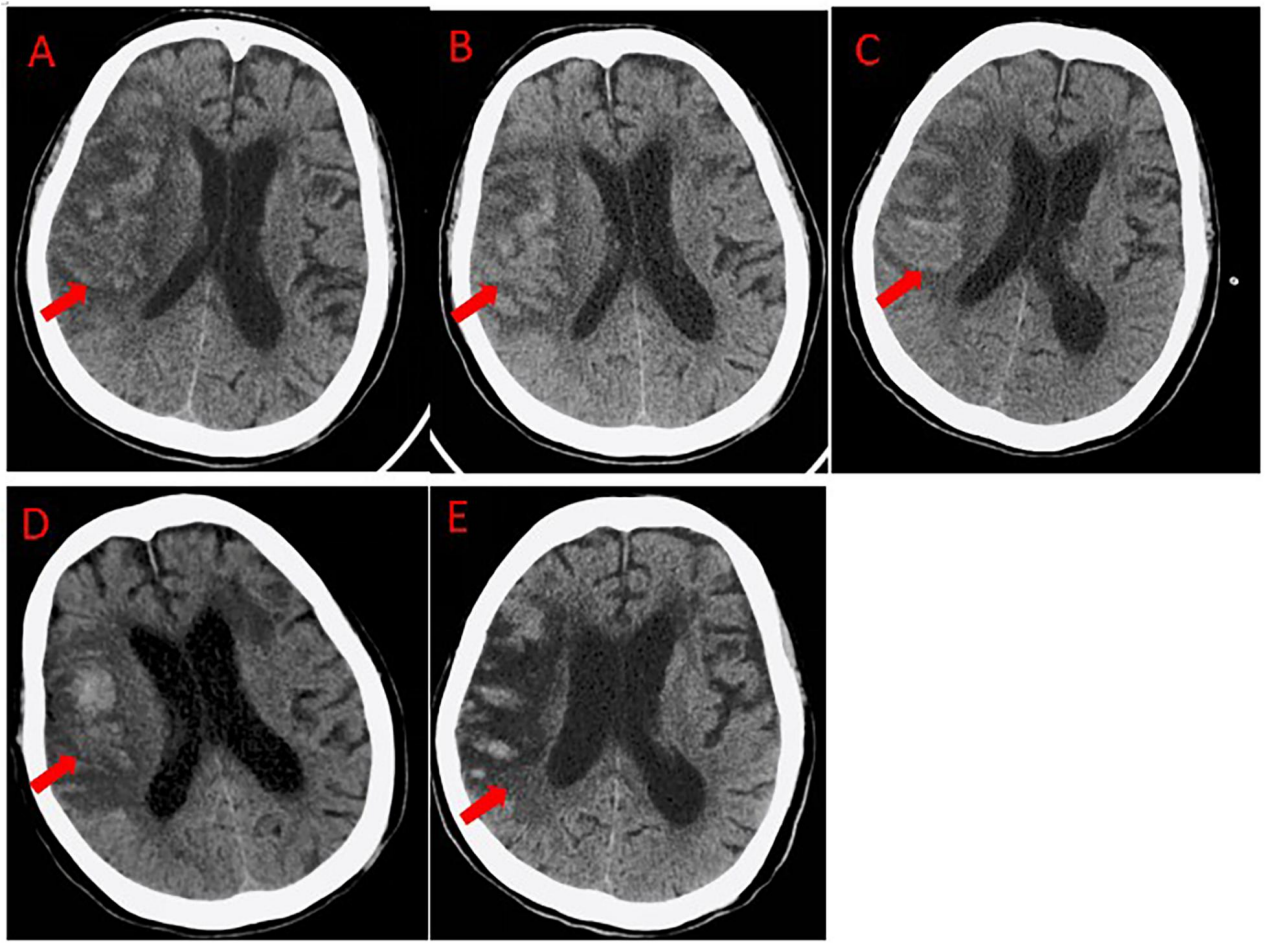
Non-contrast head CT images. **(A)** Day 4 after cerebral thrombectomy: initial development of patchy high-density areas (arrows), indicative of early hemorrhagic transformation. **(B)** Day 6 after cerebral thrombectomy: progressive expansion and coalescence of the hemorrhage (arrows). **(C)** Day 12 after cerebral thrombectomy: slight reduction in the size and density of the hemorrhage (arrows). **(D)** Day 17 after cerebral thrombectomy (pre-pulmonary intervention): re-expansion of the hemorrhage (arrows). **(E)** Day 7 after pulmonary thrombectomy: stability of the intracranial hemorrhage, confirming no procedure-related exacerbation (arrows).

**Figure 2:**
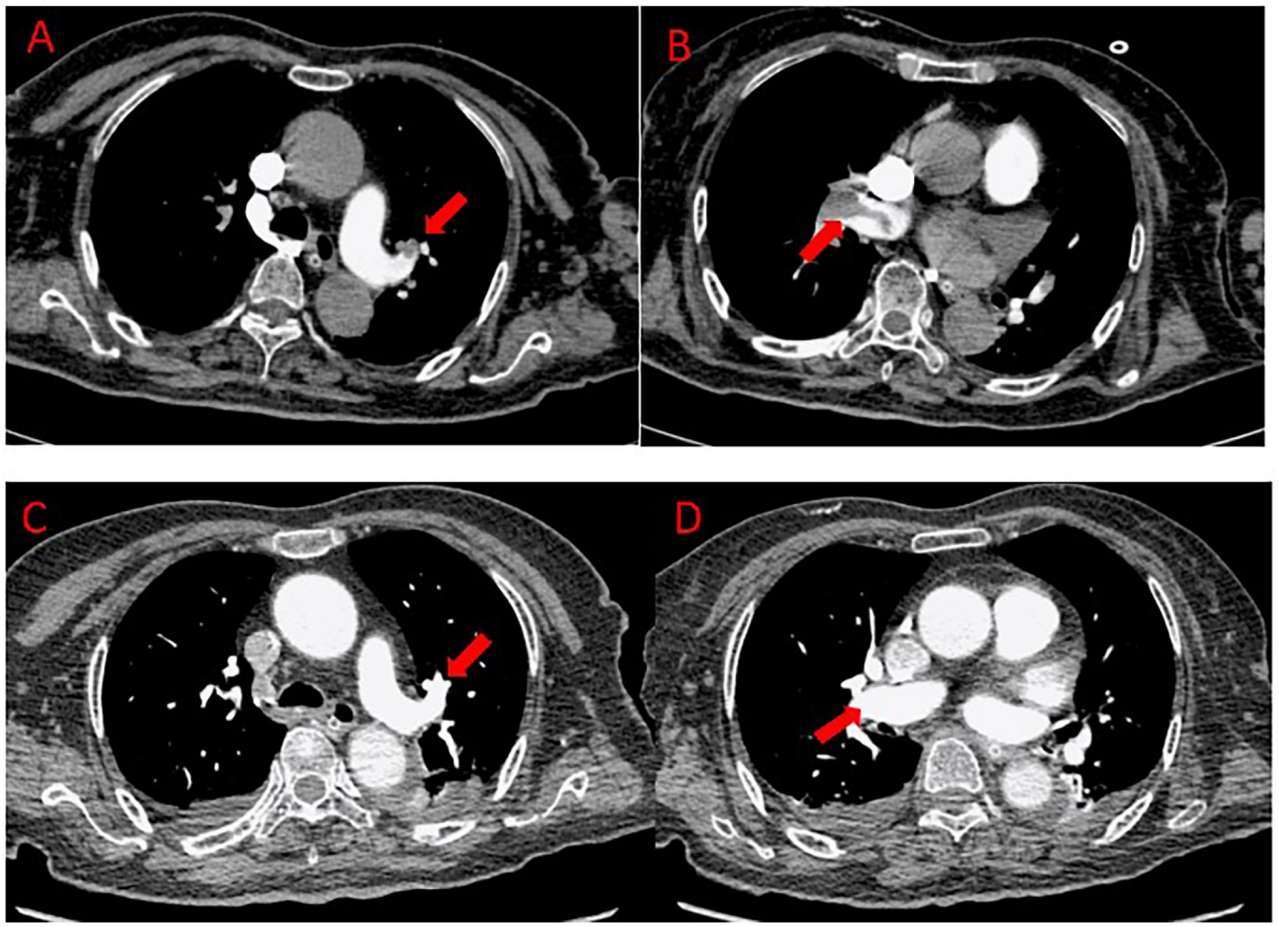
CT pulmonary angiography images. **(A,B)** Axial CTPA images on day 17 after cerebral thrombectomy (at presentation of PE): large filling defects (arrows) are seen occluding the right main pulmonary artery and its branches, diagnostic of acute massive PE. **(C,D)** Follow-up axial CTPA images on day 7 after pulmonary aspiration thrombectomy: near-complete resolution of the previously seen filling defects, indicating successful mechanical thrombus removal and restoration of pulmonary arterial flow.

On postoperative day 4, the patient became progressively somnolent. A follow-up non-contrast head CT revealed new high-density areas in the infarcted region, suggestive of hemorrhagic transformation (Figure 1A). All anticoagulants were immediately discontinued. By postoperative day 6, repeat non-contrast head CT scans showed progressive expansion of the hemorrhage (Figure 1B). During this period, all forms of antithrombotic therapy were strictly avoided. The confirmed and progressive nature of the intracranial hemorrhage rendered systemic anticoagulation and thrombolysis absolutely contraindicated. By postoperative day 12, non-contrast head CT showed slight reduction of hemorrhage (Figure 1C). However, on postoperative day 17, the patient's condition suddenly deteriorated, with acute severe dyspnea. Under nasal oxygen supplementation, pulse oxygen saturation (SpO_2_) dropped to 92% and continued to decline. Even after switching to face-mask oxygen (6 L/min), SpO_2_ further decreased to 85%, accompanied by severe hypotension (85/57 mmHg), requiring aggressive vasopressor support with norepinephrine (up to 0.8 μg/kg/min) and dopamine (up to 6 μg/kg/min) to maintain a systolic blood pressure above 90 mmHg. The patient developed refractory hypoxemia and circulatory failure, requiring vasopressor support to maintain hemodynamic stability. Laboratory results revealed a sharp rise in D-dimer to 11.78 mg/L. Echocardiography demonstrated severe right heart strain with failure, evidenced by a pulmonary artery systolic pressure of 65 mmHg, a reduced TAPSE of 12 mm, and right atrial enlargement (55 × 50 mm). Lower extremity duplex ultrasonography confirmed thrombosis in the left femoral and popliteal veins. Emergency CT pulmonary angiography (CTPA) demonstrated multiple filling defects in both pulmonary arteries, with large thrombi in the right main pulmonary artery and its major branches (Figures 2A,B). Simultaneous non-contrast head CT confirmed right frontotemporoparietal and basal ganglia infarction with hemorrhagic transformation, showing increased hemorrhagic extent (Figure 1D).

Given the hemodynamic collapse and imaging findings, the patient was diagnosed with life-threatening high-risk (massive) pulmonary embolism concurrent with progressive intracranial hemorrhage—posing a lethal therapeutic paradox: systemic thrombolysis or anticoagulation was urgently indicated for PE, yet absolutely contraindicated due to evolving intracranial bleeding. After an emergency multidisciplinary discussion, the team decided to proceed with emergency intubation under general anesthesia and a “zero-drug” purely mechanical endovascular strategy. The interventional plan included bilateral lower-extremity venography, inferior vena cava (IVC) venography, pulmonary angiography, mechanical aspiration thrombectomy, and IVC filter placement. Considering the ongoing intracranial hemorrhage, no heparin or thrombolytic agents (e.g., urokinase) were administered at any stage. Intraoperative angiography revealed large thrombi in the left deep veins and bilateral pulmonary arteries. Using a large-bore sheath (20 F) and thrombectomy system (CX20), extensive mechanical aspiration thrombectomy was performed on both pulmonary arteries, successfully removing massive thrombi. Acute hypotension ensued, primarily attributable to obstructive shock from massive pulmonary embolism. This hemodynamic instability may have been further aggravated by reperfusion-related pulmonary vascular injury and transient right ventricular dysfunction following rapid thrombus removal. It was promptly managed with vasopressor support, leading to stabilization. An IVC filter was placed to prevent recurrent embolism from lower-extremity thrombi. Following mechanical thrombectomy, the patient was transferred to the intensive care unit. Her immediate postprocedural blood pressure was 92/65 mmHg, requiring continued support with norepinephrine (0.8 µg/kg/min) and dopamine (6 µg/kg/min). A marked improvement in oxygenation was observed, with the arterial partial pressure of oxygen (PaO₂) increasing from 64 mmHg pre-procedure to 84 mmHg post-procedure. The most direct hemodynamic evidence of procedural efficacy was a significant reduction in the pulmonary artery systolic pressure, measured invasively during the procedure, from 67 mmHg pre-thrombectomy to 28 mmHg immediately post-thrombectomy. This confirmed substantial relief of the right ventricular pressure overload. Due to the high doses of vasopressors required at the peak of shock, weaning was gradual; all vasopressor support was successfully discontinued by postoperative day 3. The procedural blood loss and hemodilution were reflected in a drop in hemoglobin (from 138 g/L to 93 g/L) and albumin (from 38.1 g/L to 19.1 g/L) levels, which were managed with transfusion of 2 units of packed red blood cells and 40 g of albumin. The patient's respiratory function improved steadily, allowing for extubation on postoperative day 2.

Follow-up CT pulmonary angiography on postoperative day 7 showed that most pulmonary thrombi had been removed and blood flow significantly improved (Figures 2C,D). Repeat non-contrast head CT confirmed that the intracranial hemorrhage had not worsened after the procedure (Figure 1E), demonstrating the safety of the purely mechanical strategy. The patient's condition gradually stabilized, and she was transferred to the rehabilitation department for continued treatment and the patient was ultimately discharged in a stable condition with significant neurological improvement. She was alert, able to eat independently, and had regained partial motor function (left upper limb: grade 3; left lower limb: grade 4). Her cardiopulmonary function had returned to baseline.

## Discussion

The scenario of “progressive hemorrhagic transformation after ischemic stroke complicated by high-risk pulmonary embolism” presents clinicians with an extreme therapeutic dilemma. On one hand, active intracranial hemorrhage constitutes an absolute contraindication to any form of anticoagulation or thrombolysis; on the other hand, circulatory collapse caused by pulmonary embolism urgently requires reperfusion therapy to save the patient's life. This paradox is recognized in current major guidelines as one of the most challenging clinical situations.

Authoritative guidelines clearly state that active intracranial bleeding is an absolute contraindication to both systemic and catheter-directed thrombolysis (4, 5). In patients with high-risk pulmonary embolism where thrombolysis is contraindicated, guidelines consistently recommend pure mechanical thrombectomy or surgical embolectomy as alternative reperfusion strategies (5). The clinical decision-making in this case strictly adhered to this core principle. Notably, while guidelines recommend mechanical thrombectomy, they provide little detail regarding its implementation and safety under conditions of “zero anticoagulation.” The value of this case lies not only in following the broad guideline recommendation but also in providing practical technical details and safety validation through successful execution.

The primary risk of performing interventional procedures in the context of progressive post-stroke hemorrhage is iatrogenic exacerbation of bleeding. The successful implementation of a“zero anticoagulation/zero thrombolysis” strategy in this case relied on two critical premises. First, precise anatomical safety assurance: preoperative echocardiography ruled out right-to-left shunts such as a patent foramen ovale (PFO). This assessment was crucial, ensuring that any thrombi dislodged during procedures (e.g., lower-extremity venography, pulmonary artery aspiration) remained confined to the right heart and pulmonary circulation, thereby preventing paradoxical embolism into the systemic circulation (e.g., cerebral paradoxical embolism). This fundamentally eliminated the anatomical basis for procedure-induced new cerebral infarction. Second, The procedure was performed using the domestically developed Tendvia® system. A 20 F large-lumen aspiration catheter (outer diameter: 6.7 mm, inner diameter: 6.0 mm) with an ultra-soft distal tip was advanced over a 0.035-inch guidewire. This aligns with the established principle that large-bore aspiration devices, such as the FlowTriever system, can achieve rapid thrombus extraction and significantly shorten procedural time compared to pharmacologic thrombolysis (6). Although our center utilized the Tendvia® system, its design philosophy and mechanism of action are analogous to these high-efficiency aspiration platforms (7). Throughout the procedure, the sheath was continuously flushed with normal saline; no heparin or other anticoagulant was administered. Manual aspiration was executed using a high-volume (60 mL) locking syringe. The core thrombectomy phase was efficiently completed within approximately 15 min (total procedure time: 72 min). This shorter procedure duration theoretically reduces the window for device-related thrombosis under anticoagulant-free conditions. This purely mechanical, aspiration-based approach also minimizes repeated mechanical manipulation or pharmacologic injury to the vessel wall, thereby maximizing procedural safety. Third, Notably, and consistent with the theoretical concern, some fresh thrombi were aspirated intraoperatively, which validated the risk of acute device-related thrombosis in the absence of systemic anticoagulation. Crucially, these thrombi were limited in amount and were promptly and effectively cleared by the high-flow aspiration system, demonstrating the system's capability to manage this inherent risk and maintain procedural success.” Importantly, they did not impede successful reperfusion of the pulmonary artery trunk nor compromise final hemodynamic stabilization.

Recent case reports have described different treatment strategies for patients facing a similar therapeutic dilemma. These include low-dose catheter-directed thrombolysis combined with mechanical thrombectomy, as reported by Poster (8) and Hoffman (9), mechanical thrombectomy performed under systemic anticoagulation as described by Yash (10) and William (11), and surgical embolectomy supported by VA-ECMO without anticoagulation, as reported by Brian (12). While these approaches may be appropriate in selected clinical settings, each carries inherent limitations when active or progressive intracranial hemorrhage is present.

The present case highlights a distinct and clinically important alternative. In a patient with life-threatening high-risk pulmonary embolism and progressive intracranial hemorrhage—where both anticoagulation and thrombolysis are absolutely contraindicated—purely mechanical, percutaneous pulmonary thrombectomy performed under a strict “zero anticoagulation/zero thrombolysis” strategy achieved rapid hemodynamic stabilization. After careful exclusion of intracardiac right-to-left shunts, this minimally invasive approach provided effective reperfusion while avoiding the additional risks associated with systemic anticoagulation, thrombolytic therapy, surgical intervention, or ECMO support. This case underscores that, in carefully selected patients, zero-drug mechanical thrombectomy may represent a feasible and life-saving option in one of the most challenging clinical scenarios encountered in contemporary practice.

## Conclusion

This case demonstrates that a purely mechanical aspiration thrombectomy strategy can be a lifesaving therapeutic option for patients with a massive pulmonary embolism when anticoagulation is contraindicated due to concurrent intracranial hemorrhage.

## Data Availability

The datasets presented in this study can be found in online repositories. The names of the repository/repositories and accession number(s) can be found in the article/Supplementary Material.

## References

[1] Kovács KB Bencs V Hudák L Oláh L Csiba L. Hemorrhagic transformation of ischemic strokes. Int J Mol Sci. (2023) 24(18):14067. 10.3390/ijms241814067 37762370 PMC10531605

[2] Honig A Percy J Sepehry AA Gomez AG Field TS Benavente OR. Hemorrhagic transformation in acute ischemic stroke: a quantitative systematic review. J Clin Med. (2022) 11(5):1162. 10.3390/jcm11051162 35268253 PMC8910828

[3] Furie KL Kasner SE Adams RJ Albers GW Bush RL Fagan SC Guidelines for the prevention of stroke in patients with stroke or transient ischemic attack: a guideline for healthcare professionals from the American Heart Association/American Stroke Association. Stroke. (2011) 42(1):227–76. 10.1161/STR.0b013e3181f7d043 20966421

[4] Salinas P Cid Álvarez AB Jorge Pérez P Vázquez-Álvarez ME Jurado-Román A Juárez M Catheter-directed interventions in acute pulmonary embolism: position statement of SEC-interventional cardiology association/SEC-ischemic heart disease and acute cardiovascular care association/SEC-working group on pulmonary hypertension. Rev Esp Cardiol (Engl Ed). (2025) 78(3):239–51. 10.1016/j.rec.2024.09.011 39566820

[5] Meyer G Becattini C Geersing GJ Harjola VP Huisman MV Humbert M 2019 ESC guidelines for the diagnosis and management of acute pulmonary embolism developed in collaboration with the European Respiratory Society (ERS). Eur Heart J. (2020) 41(4):543–603. 10.1093/eurheartj/ehz405 31504429

[6] Wible BC Buckley JR Cho KH Bunte MC Saucier NA Borsa JJ. Safety and efficacy of acute pulmonary embolism treated via large-bore aspiration mechanical thrombectomy using the inari FlowTriever device. J Vasc Interv Radiol. (2019) 30(9):1370–5. 10.1016/j.jvir.2019.05.024 31375449

[7] Lu X Liu S Chen C Chen J Xu S Luo Y Efficacy analysis of the TendviaTM pulmonary artery stent thrombectomy system in the treatment of intermediate-to high-risk pulmonary embolism. Front Cardiovasc Med. (2025) 12:1685294. 10.3389/fcvm.2025.1685294 41163960 PMC12558895

[8] Rojas-Marte G Kinno M Patel M Olusanya A Solanki P. Use of pharmacomechanical thrombectomy in a patient with acute pulmonary embolism and imminent hemodynamic collapse at very high risk for cerebral hemorrhage. J Am Coll Cardiol. (2017) 69(11 Suppl):2413. 10.1016/S0735-1097(17)35802-3

[9] Hoffman C Govsyeyev N Siada SS Jacobs DL. Massive pulmonary embolism with cardiac arrest during routine tibial bypass surgery. Ann Vasc Surg. (2021) 73:509.e15–e19. 10.1016/j.avsg.2020.11.002 33333184

[10] Varma Y Shah R Shah Y Patel BA Halabi AR Aloka F Triple trouble: large right cerebral stroke with hemorrhagic transformation, DVT, and massive pulmonary embolism. J Am Coll Cardiol. (2023) 81(8 Suppl):3128. 10.1016/S0735-1097(23)03572-6

[11] Ciurylo W. Submassive pulmonary embolism in the setting of intracerebral hemorrhage: a case of suction thrombectomy. Cureus. (2022) 14:32432. 10.7759/cureus.32432 PMC9833621 36644103

[12] Ayers B Wood K Cameron S Marinescu M Bjelic M Barrus B Surgical pulmonary embolectomy with no systemic anticoagulation for patient with recent stroke. Ann Thorac Surg. (2020) 110(6):e493–5. 10.1016/j.athoracsur.2020.04.033 32473129 PMC7669635

